# Burden of excess mortality after implementation of the new kidney allocation system may be borne disproportionately by middle-aged recipients

**DOI:** 10.1371/journal.pone.0210589

**Published:** 2019-01-24

**Authors:** Catherine R. Butler, James D. Perkins, Christopher K. Johnson, Christopher D. Blosser, Ramasamy Bakthavatsalam, Nicolae Leca, Lena Sibulesky

**Affiliations:** 1 Department of Medicine, Division of Nephrology, University of Washington Medical Center, Seattle, WA, United States America; 2 Department of Surgery, Division of Transplant Surgery, University of Washington Medical Center, Seattle, WA, United States America; Imperial College Healthcare NHS Trust, UNITED KINGDOM

## Abstract

Under the new kidney allocation system (KAS), implemented in 2014, the distribution of the best quality donor kidney grafts shifted between age groups, but it is unclear whether this change translates to meaningful differences in post-transplant outcomes. We conducted a retrospective cohort study of 20,345 deceased donor kidney transplant recipients before and 4,605 recipients after implementation of the KAS using data from the United Network of Organ Sharing. Overall, two-year mortality was greater among recipients in the post-KAS era compared with the pre-KAS era (6.31% vs 5.91% respectively, [p = 0.01]), and two-year graft loss was not significantly different between eras (9.95% and 9.65%, respectively [p = 0.13]). In analysis stratified by age group (18–45, 46–55, 56–65, and ≥66 years), relative risk of mortality was 1.48 (95% confidence interval [CI] 1.09–1.98) among recipients 46–55 years old and 1.47 (95% CI 1.18–1.81) among recipients 56–65 years old. Relative risk of all-cause graft loss was 1.43 (95% CI 1.20–1.70) among recipients 56–65 years old. There were no significant differences in relative risk of mortality or graft loss associated with the KAS era among other age groups. After adjustment for recipient characteristics and characteristics of the changing donor pool, relative risk of two-year mortality and graft loss associated with the post-KAS era was attenuated for recipients aged 46–55 and 56–65 years, but remained statistically significant. In this early analysis after implementation of the KAS, there is suggestion that increased risk of mortality and graft loss may be disproportionately borne by middle-aged recipients, which is only partially accounted for by changes in recipient and donor characteristics. These findings signal a need to continue to monitor the effects of the KAS to ensure that allocation practices both maximize utility of the kidney graft pool and respect fairness between age groups.

## Introduction

Deceased donor kidney grafts available for transplantation in patients with end-stage renal disease are a scarce and precious societal resource. For this reason, since the late 1960s, when regional organ-sharing programs began to coalesce, the process and criteria by which donor grafts are allocated among potential recipients has been under public scrutiny [1]. In 2000, the US federal government issued the Organ Procurement and Transplantation Network (OPTN) “Final Rule,” requiring an allocation system to balance considerations of both efficient use of donor organs and equitable patient access to transplantation [2]. This triggered an in-depth internal review of the national deceased donor kidney allocation system and call to improve both the overall life-years gained from the pool of donor grafts and also to address several observed disparities in access to transplantation. After more than a decade of discussion, the new kidney allocation system (KAS) was implemented in 2014. A waitlist—which promotes equity by giving priority to candidates who have been listed the longest—continues to be the backbone of the kidney allocation system. In order to address recognized disparities in access to transplantation among patients with a high degree of sensitization to donor antigens and those who are listed late after starting dialysis, these groups are now given special priority on the deceased donor waitlist [3, 4]. Because these groups are at higher risk of poor post-transplant outcomes, the transplant community acknowledged that these particular modifications to the allocation system—though intended to improve fairness—would nonetheless sacrifice some utility in terms of overall life-years gained from the limited organ supply [4]. In contrast, a concurrent “longevity matching” strategy aims to improve the overall utility of the limited organ supply. Recipients with the longest expected post-transplant longevity are now preferentially offered those deceased donor grafts that are expected to last the longest, thus extending the duration of graft survival among healthier recipients and reducing the need for repeat transplantation [4, 5]. While longevity matching only explicitly involves the 20% of kidney grafts with the highest quality as estimated by the kidney donor profile index (KDPI) and the 20% of recipients with the longest estimated post-transplant survival (EPTS), the types and characteristics of grafts remaining in the donor pool that are allocated to the remaining 80% of recipients will inevitably differ.

The ethical appropriateness of limiting transplantation for older patients has long been debated by clinicians, policy-makers, and ethicists [6–12], and while discussion is ongoing, the US has largely rejected explicit age-based allocation practices [8, 13, 14]. However, because recipient age is a prominent component of the EPTS score used for longevity matching, adults over 55 years old are effectively excluded from receiving the highest quality grafts [15]. There appears to be small change in access to transplantation among older adults in the first few years after implementation of the KAS [5, 16]; however, while equity in access to transplant remains important, equity in treatment quality also has moral implications. It is not clear whether changes in the distribution of high-quality grafts after implementation of the new kidney allocation system differentially impact the post-transplant outcomes of different age groups.

The complex and interrelated effects of longevity matching in combination with other changes to the KAS are difficult to predict, making retrospective review critical in assessing whether we are achieving the intended goals of improved utility and equity. Now, more than three years after initial implementation, we have an opportunity to compare early patterns in recipient outcomes before and after implementation of the new KAS. Here, we describe two-year graft and recipient survival among different age groups before and after implementation of the new KAS.

## Methods

### Study population and data source

We conducted a retrospective cohort analysis of recipients in the United Network for Organ Sharing (UNOS) Standard Analysis and Research file. United States donor and recipient data originated from the OPTN data release 12/1/2017, which was collected through 9/30/2017. All data were fully anonymized before we accessed it, and the IRB waived the requirement for informed consent. This study was exempt from review as approved by the University of Washington Institutional Review Board.

Two groups of kidney transplant recipients were defined by whether they underwent transplantation before institution of the KAS between 12/5/2012 and 12/4/2014 (termed the “pre-KAS” group) or after institution of the KAS between 5/5/2015 and 9/30/2015 (termed the “post-KAS” group). Those transplanted within six months after institution of the KAS were excluded to limit the effect of the initial bolus of recipients who were highly sensitized to donor antigens and/or had spent an extended period of time on dialysis [17]. Recipients were excluded if they were younger than 18 years old. The resulting cohort included 20,345 patients who received a deceased donor kidney transplant before, and 4,605 patients who received transplant after institution of the KAS. These groups were further stratified by age category (18–45, 46–55, 56–65, and ≥ 66 years). Age categories were selected to optimize granularity within the limits of a relatively small sample size. There were no significant differences for the outcomes between age groups 18–30 and 31–45 years old, so these groups were combined for the final analysis.

### Outcomes

Data for all patients were collected for 790 days after transplantation. This duration represents a two-year follow-up period and an additional 30 days to account for late filing of Scientific Registry of Transplant Recipients forms. The primary outcomes were all-cause recipient mortality and all-cause graft loss over two years of follow up.

### Covariates

Using UNOS data from the date of transplantation, we ascertained age, sex, race, panel reactive antibody (PRA), receipt of prior kidney transplant, diabetes status, peripheral vascular disease status, years spent on dialysis, and years spent on the deceased donor kidney waitlist. Using UNOS donor data, we ascertained donor KDPI, hepatitis C virus (HCV) serostatus, cold ischemia time, and public health service (PHS) increased risk status. There were 88 missing values for cold ischemia time, which were imputed by linear regression using distance between donor and recipient and local, regional, or national sharing of donor data. Ten missing values for race were assigned the majority category (white). We collected data on induction agent from UNOS one-month follow-up reports. We ascertained cause of death reported through UNOS transplant recipient follow-up forms.

### Statistical analysis

We described the characteristics of recipients at the time of transplantation with percentages for categorical variables and mean (standard deviation [SD]) for continuous variables. P < 0.05 was considered significant for all statistical analyses. We used Cox proportional hazard analysis and log-rank test to produce Kaplan-Meier survival curves to detect a difference in death and graft loss between groups transplanted before and after implementation of the new KAS over two years of follow-up. The number of variables included in multivariable analysis were restricted to avoid over-fitting, and chosen to represent recipient factors that varied between the pre and post-KAS eras as well as changes in characteristics of the donor graft pool between these time periods. Univariable sensitivity analysis was performed using ranges of PRA (0–20, 21–80, 81–98, and ≥99), and only PRA ≥99 reached statistical significance for association with the primary outcomes. We excluded PRA ≥99 from the final multivariable model due to multicollinearity with receipt of a previous transplant. Similarly, we excluded diabetes due to multicollinearity with peripheral arterial disease. Correlation between years spent on the deceased donor waitlist and years spent on dialysis was 0.55, so both were included in the multivariable analysis. Because changes in graft KDPI and cold ischemic time were anticipated effects of the new KAS [18], they were considered to be related to the “KAS era” variable for this analysis and were not included separately. Ultimately, we included sex, race/ethnicity, peripheral vascular disease, previous transplant, years on the deceased donor waiting list, years on dialysis, graft PHS increased risk status, and graft HCV positive serostatus in the multivariable analysis. Analyses were conducted using JMP Pro 13.1.0 (SAS Institute Inc., Cary, NC) statistical software.

## Results

### Characteristics

Deceased donor kidney transplant recipients were more often black and/or Hispanic and fewer had diabetes in the post-KAS era compared with the pre-KAS era (Table 1). PRA≥99 was more common in the post vs. pre-KAS era for all age groups, and was especially common among younger recipients. Recipients spent longer periods of time on dialysis and on the waiting list prior to transplantation in the post-KAS era, and patients in older age groups spent less time on dialysis and on the deceased donor waitlist on average than other groups. A greater proportion of recipients had received a prior kidney transplant in the post-KAS era compared with the pre-KAS era. Rates of diabetes were lower in the post-KAS era among those aged 18–45 years, but higher for all other age groups. Rates of peripheral arterial disease were lower in the post-KAS era for all age groups except those ≥66 years old.

**Table 1:** Recipient and donor characteristics pre and post new kidney allocation system (KAS).

	Overall		18–45 years old		46–55 years old		56–65 years old		≥66 years old	
	Pre-KAS	Post-KAS	Pre-KAS	Post-KAS	Pre-KAS	Post-KAS	Pre-KAS	Post-KAS	Pre-KAS	Post-KAS
	n = 20345	n = 4605	n = 5028	n = 1477	n = 4913	n = 1063	n = 6366	n = 1305	n = 4038	n = 760
**Recipient characteristics**										
Female sex, %	39	40.4	41.6	43.4	37.4	38.7	39.7	39.2	36.4	39
Race/Ethnicity, %										
Asian	7.2	7.1	7.4	6.8	6.1	6.4	7.6	7.5	7.7	8
Black	32	36.4	38	38.6	35.4	41.3	30.3	36.3	22.7	25.3
Hispanic	16.4	17.8	19.1	22.3	17.2	17.9	16.3	16	12.2	11.8
Other	2.1	2.4	2	2.7	2.2	2.2	2.2	2.2	1.5	2.1
White	42.4	36.4	33.5	29.5	39	32.3	43.5	37.9	55.9	52.8
PRA ≥99, %	2.4	12.7	3.7	15.8	2.9	16.1	1.8	10.4	1.1	5.8
Prior kidney transplant, %	12.4	16.2	20.9	23.2	13.5	18.9	9.1	12	5.7	5.9
On dialysis, %	77.1	90.6	83.1	92.2	78	91.2	75.7	90.9	70.6	86.2
Years on dialysis (mean [SD])	3.9 (3.5)	5.1 (3.6)	4.5 (3.7)	5.4 (3.7)	4.2 (3.8)	5.5 (3.8)	3.7 (3.3)	5.0 (3.6)	3.0 (2.9)	4.2 (3.2)
Years on deceased donor waitlist (mean [SD])	3.3 (2.3)	5.4 (3.4)	3.5 (2.4)	5.6 (3.5)	3.5 (2.4)	5.9 (3.5)	3.2 (2.2)	5.3 (3.4)	3.0 (2.0)	4.7 (3.0)
Diabetes (%)	38.1	35.6	18.1	12.8	34.7	35.5	49.6	51.1	48.7	52.6
Peripheral Vascular Disease, %	10.2	9.1	5	4.2	9.1	8.8	13.3	12	13.2	14.3
**Donor characteristics**										
KDPI category, %										
0–20	18.8	21.3	25.7	46.7	19.7	14.4	16.7	7.3	12.4	5.4
21–50	33.6	32.8	40.3	27.9	36.7	38.6	31.1	35.6	25.3	29.5
51–85	37.5	37.3	31.6	23.8	37.3	41.4	39.3	44.4	42.2	45.9
85–100	10.2	8.6	2.4	1.6	6.4	5.6	13	12.8	20	19.2
HCV Serostatus Positive, %	5	6.7	2.4	2.2	5.2	6.2	7.9	13	3.2	5.4
Cold Ischemia time, hr (mean [SD])	17.3 (8.6)	18.3 (8.9)	16.9 (8.5)	17.6 (8.8)	17.1 (8.5)	18.3 (8.7)	17.4 (8.8)	18.6 (8.9)	17.8 (8.8)	19.4 (9.11)
PHS increased risk, %	16.2	21.4	16	25.3	16.6	20.2	16.9	20.6	14.7	16.5

Abbreviations: Panel reactive antibody (PRA), standard deviation (SD), kidney donor profile index (KDPI), hepatitis C virus (HCV), Public health service (PHS)

Donor kidneys in the post-KAS era were more often classified as PHS increased risk and had a longer average cold-ischemia time. A greater percentage of donor kidneys among recipients aged 18–45 years had a KDPI of 0–20 in the post-KAS era compared with the pre-KAS era (46.72% vs. 25.74%, respectively), and the percentage of donor kidneys with KDPI 0–20 was lower in the post-KAS era in all other age groups (14.4% vs. 19.7%, respectively, among those aged 46–55 years, 7.3% vs. 16.7% among those aged 56–65 years, 5.6% vs. 12.7% among those aged 66–75 years). A greater proportion of donor kidneys had a KDPI of 21–85 in the post-KAS era among all age groups except those aged 18–45 years.

### Outcome

**Fig 1:**
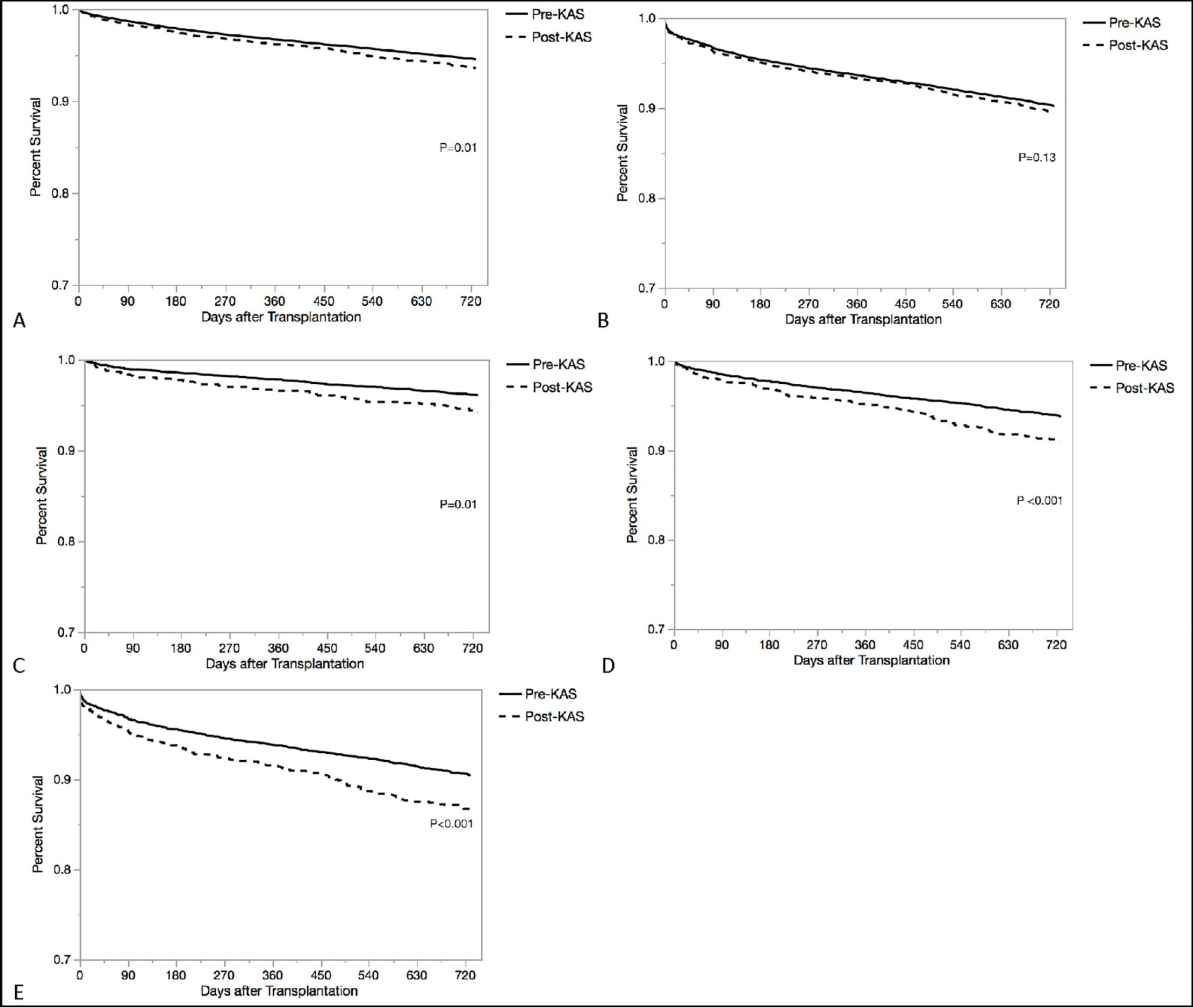
Kaplan-Meier curves for two-year survival comparing recipients transplanted pre-KAS and post-KAS, respectively. P-values were calculated by log-rank test. **A**. Recipient survival in the overall cohort; **B**. Graft survival in the overall cohort; **C**. Recipient survival among those aged 46–55 years; **D**. Recipient survival among those aged 56–65 years; **E**. Graft survival among those aged 56–65 years.

Overall, two-year patient mortality was greater among kidney transplant recipients in the post-KAS era compared with the pre-KAS era (6.31% post-KAS vs 5.91% pre-KAS [p = 0.01]) (Table 2, Fig 1). In analysis stratified by age group, two-year patient mortality was significantly greater in the post-KAS era compared with the pre-KAS era among patients aged 46–55 years (5.27% vs. 3.79% [p = 0.01]) and 56–65 years (8.28% vs. 6.06% [p<0.001]). Overall, two-year all-cause graft loss was not statistically different between the post and pre-KAS eras (9.95% and 9.65% [p = 0.13]). However, two-year all-cause graft loss was significantly greater in the post-KAS era among recipients aged 56–65 years (12.72% vs. 9.41% [p<0.001]) (Table 2). The most frequently reported causes of mortality were cardiovascular death and infection-related death (S2 Table). The rate of cardiovascular death was greater for all age groups after implementation of the KAS compared with before the KAS except among those 46–55 years old, and the rate of death related to infection was greater for all age groups except those 18–45 and ≥66 years old. Use of anti-thymocyte globulin for induction therapy was more common, and use of other agents less common, among all age groups in the post-KAS era compared with the pre-KAS era (S2 Table).

**Table 2:** Unadjusted 2-year recipient mortality and graft loss.

	pre-KAS	post-KAS	p-value
2-year recipient mortality, %			
overall	5.91	6.31	0.01
18–45 y	1.79	2.17	0.35
46–55 y	3.79	5.27	0.01
56–65 y	6.06	8.28	<0.001
≥ 66 y	10.35	10	0.93
2-year all-cause graft loss, %			
overall	9.65	9.95	0.13
18–45 y	7.52	6.84	0.57
46–55 y	8.22	8.37	0.87
56–65 y	9.41	12.72	<0.001
≥ 66 y	14.41	13.42	0.74

P-value was calculated from Kaplan-Meier survival curves using log-rank test.

Abbreviations: Kidney allocation system (KAS). Years (y)

### Adjusted analyses

In univariable analysis, relative risk of two-year mortality was 1.48 (95% confidence interval [CI] 1.09–1.98) among patients 46–55 years old and 1.47 (95% CI 1.18–1.81) among patients 56–65 years old in the post-KAS era compared with the pre-KAS era (Table 3). The relative risk of two-year all-cause graft loss was 1.43 (95% CI 1.20–1.70) among patients 56–65 years old in the post-KAS era compared with the pre-KAS era (Table 4). There was no statistically significant difference in risk associated with the post-KAS era for other age groups. In multivariable analyses, the relative risk of two-year mortality among recipients aged 46–55 years (RR 1.38 95% CI 1.01–1.85), and among recipients aged 56–65 years (RR 1.35 95% CI 1.08–1.67) and two-year graft loss among recipients aged 56–65 years (RR 1.32 95% CI 1.11–1.57) was reduced compared to the univariable analysis, but remained significant (Tables 3 and 4).

**Table 3:** Univariable and multivariable analysis of relative risk (RR) of all-cause mortality associated with measured factors.

	Univariable analysis			Multivariable Analysis		
	RR	95% CI	P	RR	95% CI	P
**Age 18–45 years**						
** Post KAS Period**	**1.26**	**0.83–1.87**	**0.26**	**1.20**	**0.8–1.88**	**0.32**
** Recipient factors**						
Female sex*	0.93	0.64–1.32	0.69	0.98	0.71–1.47	0.93
Race/Ethnicity						
White	1.00 (ref)			1.00 (ref)		
Black	1.22	0.82–1.84	0.33	1.80	0.71–1.65	0.73
Asian	0.33	0.08–0.92	0.03	0.33	0.08–0.92	0.03
Hispanic	0.82	0.47–1.38	0.45	0.83	0.41–1.24	0.25
Other	0.73	0.12–2.40	0.66	0.65	0.11–2.15	0.54
Peripheral vascular disease	3.52	2.06–5.64	<0.001	3.64	2.2–6.02	<0.001
Previous transplant	1.26	0.83–1.87	0.27	1.19	0.75–1.89	0.46
Years on deceased donor waiting list	1.08	1.01–1.16	0.02	1.07	0.99–1.15	0.10
Years on dialysis	1.07	1.02–1.11	0.00	1.05	1.01–1.10	0.04
** Donor factors**						
PHS increased risk	0.99	0.61–1.55	0.99	0.99	0.62–1.58	0.97
HCV serostatus positive	1.78	0.63–3.93	0.25	1.70	0.69–4.20	0.25
**Age 46–55 years**						
** Post KAS Period**	**1.48**	**1.09–1.98**	**0.01**	**1.38**	**1.01–1.85**	**0.04**
** Recipient factors**						
Female sex*	0.86	0.66–1.12	0.27	0.86	0.65–1.12	0.26
Race/Ethnicity						
White	1.00 (Ref)			1.00 (Ref)		
Black	0.95	0.71–1.25	0.69	0.83	0.62–1.11	0.20
Asian	0.47	0.21–0.90	0.02	0.45	0.2–0.87	0.02
Hispanic	0.67	0.44–0.98	0.04	0.60	0.39–0.88	0.01
Other	0.65	0.2–1.56	0.37	0.59	0.21–1.60	0.30
Peripheral vascular disease	1.58	1.07–2.24	0.02	1.56	1.08–2.26	0.02
Previous transplant	1.28	0.91–1.76	0.15	1.08	0.75–1.56	0.67
Years on deceased donor waiting list	1.01	0.95–1.06	0.79	0.99	0.93–1.04	0.67
Years on dialysis	1.05	1.02–1.08	<0.001	1.06	1.03–1.09	<0.001
** Donor factors**						
PHS increased risk	1.00	0.71–1.38	0.99	0.99	0.71–1.40	0.98
HCV serostatus positive	1.24	0.72–1.99	0.41	1.22	0.73–2.05	0.45
**Age 56–65 years**						
** Post KAS Period**	**1.47**	**1.18–1.81**	**<0.001**	**1.35**	**1.08–1.67**	**0.00**
** Recipient factors**						
Female sex*	1.47	1.18–1.81	<0.001	1.35	1.08–1.67	0.00
Race/Ethnicity						
White	1.00 (Ref)			1.00 (Ref)		
Black	1.08	0.88–1.32	0.48	0.89	0.72–1.10	0.27
Asian	0.86	0.59–1.22	0.41	0.79	0.54–1.12	0.19
Hispanic	0.81	0.61–1.06	0.13	0.67	0.49–0.88	0.00
Other	0.97	0.5–1.69	0.92	0.85	0.43–1.48	0.58
Peripheral vascular disease	1.54	1.22–1.93	<0.001	1.54	1.23–1.94	<0.001
Previous transplant	1.46	1.11–1.88	0.01	1.34	1.03–1.75	0.03
Years on deceased donor waiting list	1.07	1.03–1.11	<0.001	1.05	1.01–1.09	0.02
Years on dialysis	1.08	1.06–1.11	<0.001	1.07	1.05–1.10	<0.001
** Donor factors**						
PHS increased risk	0.99	0.78–1.24	0.93	0.99	0.78–1.25	0.91
HCV serostatus positive	1.23	0.92–1.63	0.16	1.15	0.85–1.56	0.35
**Age >66 years**						
** Post KAS Period**	**1.01**	**0.79–1.28**	**0.93**	**0.98**	**0.76–1.24**	**0.86**
** Recipient factors**						
Female sex*	0.74	0.61–0.90	0.00	0.77	0.64–0.92	0.00
Race/Ethnicity						
White	1.00 (Ref)			1.00 (Ref)		
Black	0.98	0.79–1.21	0.88	0.86	0.68–1.07	0.17
Asian	0.83	0.57–1.17	0.48	0.77	0.53–1.09	0.15
Hispanic	0.81	0.6–1.08	0.16	0.66	0.48–0.89	0.01
Other	1.14	0.6–2.09	0.70	0.99	0.47–1.83	0.99
Peripheral vascular disease	1.71	1.37–2.12	<0.001	1.67	1.34–2.08	<0.001
Previous transplant	1.65	1.19–2.24	0.00	1.66	1.20–2.28	0.00
Years on deceased donor waiting list	1.06	1.02–1.11	0.00	1.04	1.01–1.09	0.04
Years on dialysis	1.06	1.04–1.09	<0.001	1.07	1.04–1.10	<0.001
** Donor factors**						
PHS increased risk	0.95	0.73–1.21	0.67	0.93	0.72–1.20	0.59
HCV serostatus positive	1.20	0.75–1.8	0.43	1.20	0.77–1.88	0.42

*Reference category: Male sex.

Note: Panel reactive antibody ≥99 was excluded because of multicollinearity with previous transplant, and diabetes was excluded because of multicollinearity with peripheral vascular disease.

Abbreviations: Kidney allocation system (KAS), Relative risk (RR), Confidence interval (CI), Public Health Service (PHS), Hepatitis C virus (HCV)

**Table 4:** Univariable and multivariable analysis of relative risk (RR) of all-cause graft loss associated with measured factors.

	Univariable analysis			Multivariable Analysis		
	RR	95% CI	P	RR	95% CI	P
**Age 18–45 years**						
**Post KAS Period**	**0.96**	**0.77–1.19**	**0.73**	**0.92**	**0.73–1.14**	**0.45**
**Recipient factors**						
Female sex*	1.25	1.04–1.49	0.02	1.28	1.07–1.54	0.01
Race/Ethnicity						
White	1.00 (Ref)			1.00 (Ref)		
Black	1.37	1.11–1.69	0.00	1.29	1.04–1.61	0.02
Asian	0.68	0.42–1.04	0.08	0.68	0.42–1.05	0.09
Hispanic	0.81	0.61–1.07	0.15	0.78	0.58–1.04	0.09
Other	1.13	0.58–1.99	0.70	1.09	0.55–1.92	0.79
Peripheral vascular disease	1.60	1.11–2.22	0.01	1.65	1.15–2.29	0.01
Previous transplant	1.33	1.08–1.62	0.01	1.35	1.09–1.66	0.01
Years on deceased donor waiting list	1.05	1.01–1.09	0.01	1.02	0.98–1.06	0.26
Years on dialysis	1.05	1.03–1.08	<0.001	1.04	1.02–1.07	<0.001
**Donor factors**						
PHS increased risk	1.05	0.83–1.32	0.67	1.08	0.85–1.35	0.52
HCV serostatus positive	1.07	0.57–1.80	0.83	1.01	0.53–1.71	0.98
**Age 46–55 years**						
** Post KAS Period**	**1.07**	**0.84–1.34**	**0.58**	**0.98**	**0.77–1.24**	**0.89**
** Recipient factors**						
Female sex*	0.95	0.79–1.14	0.59	0.96	0.80–1.16	0.68
Race/Ethnicity						
White	1.00 (Ref)			1.00 (Ref)		
Black	1.26	1.04–1.54	0.02	1.14	0.93–1.40	0.21
Asian	0.81	0.51–1.21	0.31	0.76	0.48–1.14	0.20
Hispanic	0.70	0.52–0.94	0.02	0.64	0.47–0.86	0.00
Other	0.85	0.40–1.56	0.62	0.75	0.38–1.47	0.40
Peripheral vascular disease	1.18	0.87–1.55	0.28	1.18	0.89–1.58	0.26
Previous transplant	1.14	0.89–1.44	0.30	1.12	0.88–1.43	0.35
Years on deceased donor waiting list	1.03	0.99–1.06	0.17	1.01	0.97–1.04	0.91
Years on dialysis	1.05	1.03–1.07	<0.001	1.05	1.03–1.07	<0.001
** Donor factors**						
PHS increased risk	0.92	0.72–1.17	0.52	0.93	0.73–1.19	0.57
HCV serostatus positive	1.01	0.67–1.46	0.95	0.97	0.66–1.45	0.90
**Age 56–65 years**						
** Post KAS Period**	**1.43**	**1.20–1.70**	**<0.001**	**1.32**	**1.11–1.57**	**0.00**
** Recipient factors**						
Female sex*	0.81	0.69–0.94	0.00	0.82	0.71–0.96	0.01
Race/Ethnicity						
White	1.00 (Ref)			1.00 (Ref)		
Black	1.21	1.03–1.43	0.02	1.07	0.90–1.27	0.44
Asian	0.79	0.57–1.07	0.12	0.75	0.54–1.02	0.07
Hispanic	0.97	0.78–1.19	0.77	0.83	0.67–1.04	0.11
Other	0.97	0.56–1.55	0.91	0.89	0.54–1.47	0.64
Peripheral vascular disease	1.63	1.36–1.95	<0.001	1.60	1.33–1.92	<0.001
Previous transplant	1.36	1.09–1.68	0.01	1.31	1.05–1.63	0.01
Years on deceased donor waiting list	1.04	1.01–1.07	0.03	1.01	0.98–1.05	0.99
Years on dialysis	1.07	1.05–1.09	<0.001	1.06	1.04–1.08	<0.001
** Donor factors**						
PHS increased risk	0.90	0.74–1.08	0.26	0.89	0.72–1.08	0.23
HCV serostatus positive	1.14	0.89–1.43	0.30	1.04	0.81–1.32	0.78
**Age >66 years**						
** Post KAS Period**	**0.96**	**0.78–1.18**	**0.74**	**0.90**	**0.72–1.11**	**0.32**
** Recipient factors**						
Female sex*	0.83	0.71–0.97	0.02	0.84	0.71–0.98	0.03
Race/Ethnicity						
White	1.00 (Ref)			1.00 (Ref)		
Black	1.08	0.90–1.29	0.40	0.97	0.81–1.17	0.79
Asian	0.91	0.66–1.20	0.49	0.87	0.63–1.16	0.34
Hispanic	0.87	0.68–1.12	0.28	0.75	0.58–0.96	0.02
Other	1.22	0.67–2.04	0.49	1.08	0.59–1.80	0.80
Peripheral vascular disease	1.60	1.32–1.93	<0.001	1.57	1.30–1.91	<0.001
Previous transplant	1.62	1.22–2.11	0.00	1.63	1.24–2.15	<0.001
Years on deceased donor waiting list	1.05	1.02–1.09	0.01	1.02	0.99–1.06	0.21
Years on dialysis	1.06	1.04–1.08	<0.001	1.06	1.03–1.08	<0.001
** Donor factors**						
PHS increased risk	0.88	0.71–1.10	0.27	0.87	0.70–1.09	0.23
HCV serostatus positive	1.06	0.70–1.54	0.76	1.04	0.70–1.55	0.84

*Reference category: Male sex.

Note: Panel reactive antibody ≥99 was excluded because of multicollinearity with previous transplant and diabetes was excluded because of multicollinearity with peripheral vascular disease.

Abbreviations: Kidney allocation system (KAS), Confidence interval (CI), Relative risk (RR), Confidence interval (CI), Public Health Service (PHS), Hepatitis C virus (HCV)

## Discussion

Deceased-donor kidney transplant recipients aged 46–55 and 56–65 years are nearly 50% more likely to die within two years of transplantation in the era after compared with before implementation of the KAS, and graft loss within two years is 43% more likely among recipients aged 56–65 years in the post-KAS era. The increased risk of mortality and graft loss after implementation of the new KAS is not seen in the youngest and oldest age groups.

The transplant community anticipated and accepted some increased risk of recipient mortality related to implementation of the KAS in exchange for improved access to transplantation for several patient groups that are also at higher risk of poor post-transplantation outcomes [1]. However, our findings show that although average length of time on dialysis and rate of high donor antigen sensitization were higher in all age-groups after the KAS, two-year mortality is only significantly increased among those 46–55 and 56–65 years old. Further, after adjusting for differences in measured recipient characteristics and differences in characteristics of the donor kidney pool between time periods, mortality and graft survival remain significantly worse for middle-aged recipients in the post-KAS era compared with the pre-KAS era, suggesting that influences beyond recipient factors may affect outcomes. Less frequent allocation of grafts with low KDPI and increased use of grafts with higher KDPI among older patients–anticipated effects of longevity matching–may contribute to risk of early mortality and graft loss [19, 20]. Higher KDPI has been associated with delayed graft function, risk of infection, and prolonged hospitalizations, and propensity to experience these outcomes may differ between age groups [20–25]. Graft quality may also influence aspects of peri-transplant treatment practices, including choice of induction agent, which itself may have implications for patient outcomes. We do not detect a significant difference in early post-transplantation mortality and graft survival among the oldest age group in the post-KAS era compared with the pre-KAS era. This may relate to a mortality rate that was already relatively high in the pre-KAS era as well as differing patterns in cause of death.

Overall, these results likely belie a complex interaction of changing recipient factors, changes in the types and quality of kidneys allocated to each age group, and perhaps other evolving factors in the practice of kidney transplantation. Continued analysis may bring improved insight into longer-term outcomes and causal factors. Further, the anticipated beneficial effects of longevity matching—including minimizing the need for re-transplantation—may not be realized for many years, so the ultimate balance of improved utility and fairness of the KAS is far from determined. However, this study suggests early signs of imbalance in the burden of death and graft loss that is more pronounced among recipients aged 46–55 and 56–65 years. Strength of the UNOS system lies in its clearly established allocation algorithm, rigorous data collection, and open dissemination of results. This transparency facilitates ongoing evaluation, discussion, and modification of policy and practice in response to evolving balance of equity and utility in kidney allocation [4, 26, 27].

### Limitations

This study should be interpreted with several limitations in mind. First, the recent implementation of the KAS limits both the size of the post-KAS group and follow-up time. Subsequent studies will have additional power to detect associations as well as longer-term outcomes associated with changes to the KAS. Second, there were insufficient outcome events to support more comprehensive multivariable analysis, and other factors that may be associated with recipient outcomes deserve dedicated analysis. Third, there may be residual "bolus effect" six months after implementation of the KAS, causing over-representation of patients with high PRA and longer waiting times in the post-KAS cohort [28]. Finally, our analysis is limited to recipients of deceased donor grafts, so does not capture patterns in living kidney transplantation that may be indirectly affected by the KAS.

## Conclusion

Patterns in recipient outcomes two years after implementation of the KAS suggests that middle-aged recipients may be disproportionately affected by increased risk of early mortality and graft loss. These findings raise questions of distributive justice among age groups, prompting further study to understand the cause of this disparity as well as continued work to optimize efficient use of deceased donor kidneys balanced with the ethical imperative to promote fairness in kidney allocation.

## Supporting information

S1 Table Cause of death among recipients who died within 2 years of transplantation stratified by KAS era and age group. Abbreviations: New kidney allocation system (KAS), Central vascular accident (CVA).
(DOCX)

S2 Table Induction agent stratigied by age group and KAS era. New kidney allocation system (KAS), Anti-thymocyte globulin (ATG).
(DOCX)
