# Supplementary material for: Burden of excess mortality after implementation of the new kidney allocation system may be borne disproportionately by middle-aged recipients
Source: PLoS One. 2019 Jan 24;14(1):e0210589. doi: 10.1371/journal.pone.0210589 (PMC6345464; doi:10.1371/journal.pone.0210589)
Supplement: S1 Table — Abbreviations: New kidney allocation system (KAS), Central vascular accident (CVA). (DOCX) [file pone.0210589.s001.docx]

|  |  |  |  |  |  |  |  |  |  |  |  |  |  |
| --- | --- | --- | --- | --- | --- | --- | --- | --- | --- | --- | --- | --- | --- |
|  |  | **18-45 years** | |  | **46-55 years** | |  | **56-65 years** | |  | **≥66 years** | |  |
|  |  | Pre-KAS | Post-KAS |  | Pre-KAS | Post-KAS |  | Pre-KAS | Post-KAS |  | Pre-KAS | Post-KAS |  |
|  |  |  |  |  |  |  |  |  |  |  |  |  |  |
|  | Cardiac, % | 27.9 | 33.3 |  | 27.2 | 25.9 |  | 27.7 | 28.6 |  | 20.4 | 32.1 |  |
|  | CVA, % | 2.1 | 3.0 |  | 4.3 | 7.4 |  | 4.6 | 5.4 |  | 4.7 | 2.6 |  |
|  | Infection, % | 22.1 | 21.2 |  | 14.7 | 16.7 |  | 20.2 | 24.1 |  | 17.9 | 16.7 |  |
|  | Malignancy, % | 5.7 | 3.0 |  | 10.8 | 5.6 |  | 9.6 | 10.7 |  | 10.4 | 9.0 |  |
|  | Pulmonary, % | 6.4 | 3.0 |  | 9.3 | 7.4 |  | 6.1 | 4.5 |  | 10.8 | 9.0 |  |
|  | Other, % | 35.7 | 36.4 |  | 33.7 | 37.0 |  | 31.8 | 26.8 |  | 35.7 | 30.8 |  |
|  |  |  |  |  |  |  |  |  |  |  |  |  |  |

**S1 Table. Cause of death among recipients who died within 2 years of transplantation stratified by KAS era and age group.**

Abbreviations: New kidney allocation system (KAS), Central vascular accident (CVA).
