# Supplementary material for: Burden of excess mortality after implementation of the new kidney allocation system may be borne disproportionately by middle-aged recipients
Source: PLoS One. 2019 Jan 24;14(1):e0210589. doi: 10.1371/journal.pone.0210589 (PMC6345464; doi:10.1371/journal.pone.0210589)
Supplement: S2 Table — New kidney allocation system (KAS), Anti-thymocyte globulin (ATG). (DOCX) [file pone.0210589.s002.docx]

|  |  |  |  |  |  |  |  |  |  |  |  |  |  |
| --- | --- | --- | --- | --- | --- | --- | --- | --- | --- | --- | --- | --- | --- |
|  |  | **18-45 years** | |  | **46-55 years** | |  | **56-65 years** | |  | **≥66 years** | |  |
|  |  | Pre-KAS | Post-KAS |  | Pre-KAS | Post-KAS |  | Pre-KAS | Post-KAS |  | Pre-KAS | Post-KAS |  |
|  |  |  |  |  |  |  |  |  |  |  |  |  |  |
|  | Anti-thymocyte globulin, % | 57.3 | 64.0 |  | 55.2 | 61.6 |  | 54.8 | 60.2 |  | 49.9 | 56.4 |  |
|  | Alemtuzumab, % | 17.1 | 15.4 |  | 17.1 | 16.3 |  | 15.3 | 14.4 |  | 11.1 | 10.8 |  |
|  | Basilixumab, % | 12.1 | 9.6 |  | 14.5 | 10.8 |  | 15.8 | 14.4 |  | 23.8 | 20.9 |  |
|  | Steroids only, % | 2.6 | 1.8 |  | 2.2 | 0.8 |  | 2.3 | 0.9 |  | 2.5 | 1.2 |  |
|  | Other induction agent, % | 0.9 | 0.5 |  | 0.8 | 0.8 |  | 0.8 | 0.7 |  | 0.8 | 0.7 |  |
|  | No induction, % | 10.0 | 8.5 |  | 10.2 | 9.7 |  | 11.0 | 9.3 |  | 11.9 | 10.0 |  |
|  |  |  |  |  |  |  |  |  |  |  |  |  |  |

**S2 Table. Induction agent stratigied by age group and KAS era.** New kidney allocation system (KAS), Anti-thymocyte globulin (ATG).
